# Supplementary material for: Differential PaxillinB dynamics at Dictyostelium cell-substrate adhesions
Source: Biol Open. 2025 Sep 26;14(10):bio062197. doi: 10.1242/bio.062197 (PMC12505270; doi:10.1242/bio.062197)
Supplement: Supplementary information [file biolopen-14-062197-s1.pdf]

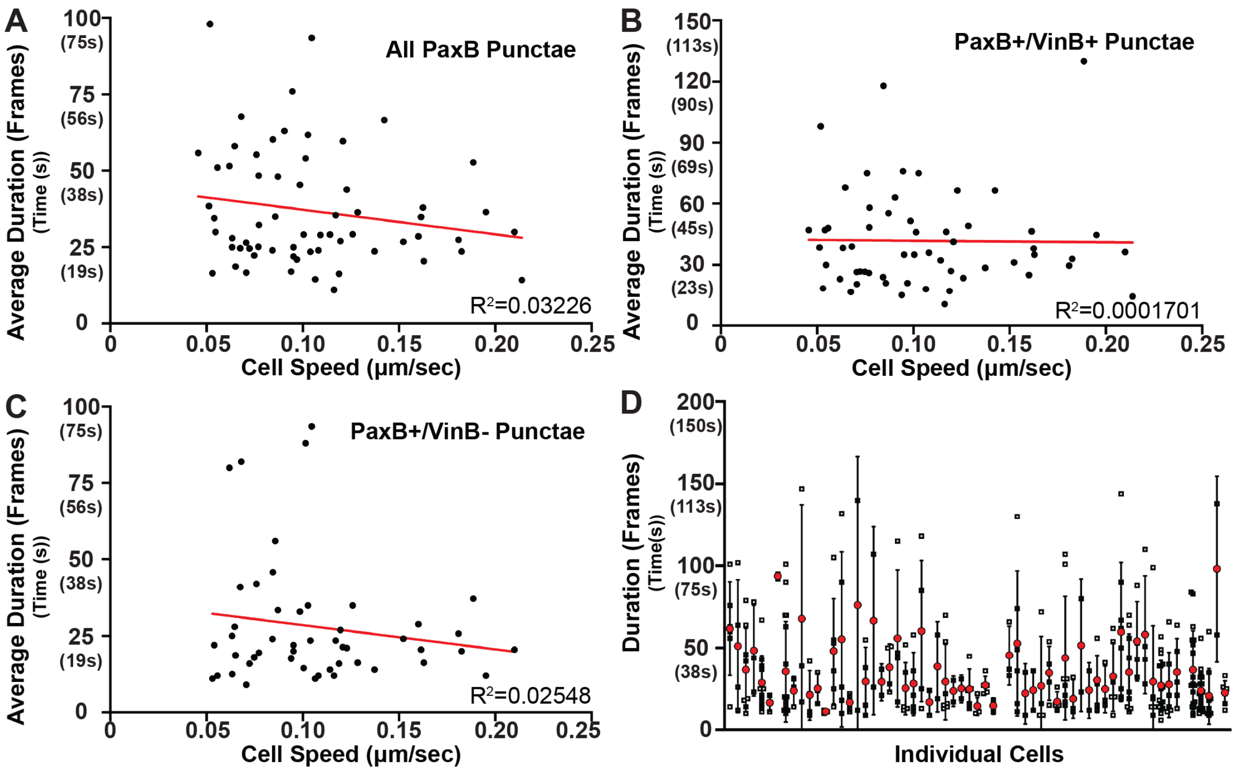

Fig. S1. *Dictyostelium* cell-substrate adhesion duration does not correlate with cell migration speed

Scatterplot graphs comparing cell migration speed to the average duration of adhesions in cells. For each cell, the average duration for all **A)** PaxillinB-positive punctae, **B)** PaxillinB+/VinculinB+ punctae or **C)** PaxillinB+/VinculinB- punctae across n = 65 (A), 57 (B), and 48 (C) cells, respectively, was correlated with cell migration speed. **D)** Distribution of individual PaxillinB punctae duration (squares) within individual cells (n= 61 cells) for cells with more than one PaxillinB punctae tracked. Each column represents duration of multiple punctae in an individual cell and the mean punctae duration is superplotted (red circles). Mean +/- SEM.

Biology Open • Supplementary information
